# Sonification for Personalised Gait Intervention

**DOI:** 10.3390/s24010065

**Published:** 2023-12-22

**Authors:** Conor Wall, Peter McMeekin, Richard Walker, Victoria Hetherington, Lisa Graham, Alan Godfrey

**Affiliations:** 1 Department of Computer and Information Sciences, Northumbria University, Newcastle upon Tyne NE1 8ST, UK; 2 Department of Nursing, Midwifery and Health, Northumbria University, Newcastle upon Tyne NE1 8ST, UK; 3 Northumbria Healthcare NHS Foundation Trust, North Shields NE29 8NH, UK; 4 Cumbria, Northumberland Tyne and Wear NHS Foundation Trust, Wolfson Research Centre, Campus for Ageing and Vitality, Newcastle upon Tyne NE4 9AS, UK; 5 Department of Sport, Exercise and Rehabilitation, Northumbria University, Newcastle upon Tyne NE1 8ST, UK

**Keywords:** gait, Parkinson’s disease, personalised rehabilitation, sonification, wearables

## Abstract

Mobility challenges threaten physical independence and good quality of life. Often, mobility can be improved through gait rehabilitation and specifically the use of cueing through prescribed auditory, visual, and/or tactile cues. Each has shown use to rectify abnormal gait patterns, improving mobility. Yet, a limitation remains, i.e., long-term engagement with cueing modalities. A paradigm shift towards personalised cueing approaches, considering an individual’s unique physiological condition, may bring a contemporary approach to ensure longitudinal and continuous engagement. Sonification could be a useful auditory cueing technique when integrated within personalised approaches to gait rehabilitation systems. Previously, sonification demonstrated encouraging results, notably in reducing freezing-of-gait, mitigating spatial variability, and bolstering gait consistency in people with Parkinson’s disease (PD). Specifically, sonification through the manipulation of acoustic features paired with the application of advanced audio processing techniques (e.g., time-stretching) enable auditory cueing interventions to be tailored and enhanced. These methods used in conjunction optimize gait characteristics and subsequently improve mobility, enhancing the effectiveness of the intervention. The aim of this narrative review is to further understand and unlock the potential of sonification as a pivotal tool in auditory cueing for gait rehabilitation, while highlighting that continued clinical research is needed to ensure comfort and desirability of use.

## 1. Introduction

Globally, the proportion of the population > 65 years (older adults) is increasing. In the UK, projections suggest that by 2046, that group will represent 25% of the population [1]. Typically, older adults face challenges due to the potential decline in gait-related mobility. That decline impacts crucial aspects of personal independence and the overall quality of life [2]. Conditions such as Parkinson’s disease (PD), stroke, and arthritis can significantly curtail an individual’s ability to freely ambulate and perform routine daily tasks unaided [3]. Functional challenges are compounded by mobility difficulties resulting in falls, which constitute the leading cause of injury-related deaths for those > 65 years [4]. Moreover, a harmful cycle due to a lack of confidence in walking due to a fear of falling can further exacerbate physical decline, highlighting a necessity for public health initiatives aimed at mitigating this escalating silent epidemic [5]. This pervasive issue underscores the critical need for practical interventions aimed at alleviating gait-related mobility issues [6].

Rehabilitation research has examined cueing, leveraging auditory, visual, and tactile cues to normalize abnormal gait patterns and improve mobility [7]. However, auditory cueing has been found to both be the most effective at improving gait and the most practical to apply in outdoor settings [8,9]. There is also an appreciation regarding cueing paradigms that one size does not fit all, i.e., there is a need to ensure personalised approaches to ensure interventions are tailored to the individual and their functional limitations at a moment in time. Furthermore, the long-term effectiveness of cueing mechanisms, such as metronomes, ultimately suffer from their lack of engagement [10]. This highlights the existing gap in personalised gait-cueing interventions that not only cater to an individual’s specific physical needs but also offer an engaging experience to promote long-term adherence.

Sonification is the methodology of using non-speech audio to communicate information [11]. Within medicine, heart rate and electrocardiograph monitors use a humble representation of sonification to audibly represent a patient’s heartbeat, alerting doctors to any potential irregularities in their cardiac cycle [12,13]. This is especially important in critical care situations, where transforming patient vital signs into audio cues allows caregivers to quickly detect and respond to any changes. Other examples showcase how sonification can be used to provide valuable enhancements in medical diagnostics and monitoring [14,15].

Applying sonification to audibly represent the rhythmic properties of a patient’s vital signs underscores its potential application in gait rehabilitation where the goal is to establish rhythmic, distinct, and harmonious walking patterns. This potential has been recognized where several implementations of sonification in gait rehabilitation have been effective in enhancing mobility, particularly for older adults who face challenges with various mobility disorders [16,17,18]. A recently conducted review emphasizes the technical and design aspects of real-time movement sonification systems relating to gait. This includes the dimensions of movement to sonify, what anatomy to track, and what motion capture methodologies to use [19]. However, while the findings of that review are valuable and insightful, it notably omits a discussion on how acoustic variables influence the valence of such rehabilitation systems. Understanding these acoustic factors is crucial, as they can significantly affect patient engagement and treatment outcomes. Additionally, the review does not delve into the potential for manipulating these acoustic variables to provide more personalised interventions to further enhance the valence and overall usability of these systems. Understanding these gaps could lead to more effective and user-friendly rehabilitation solutions, tailored to the unique needs and preferences of individuals undergoing neurological rehabilitation.

Here, we explore the concept of personalised interventions, focusing on sonification and its potential in gait rehabilitation. This review discusses ways sonification can enhance gait-related mobility, how it can be manipulated, and how it can be used to improve the landscape of gait rehabilitation.

## 2. Assessing Gait

Measuring the gait of those with physical mobility challenges is an initial step to providing effective and long-lasting rehabilitation strategies. By identifying which aspects of an individual’s gait are abnormal, pragmatic approaches can be devised [20]. Digital technologies are playing an important role within instrumented rehabilitation, objectifying gait to provide high-resolution data. For example, instrumented walkways have integrated pressure sensors that record footfall data as a person walks across the surface, generating a comprehensive representation of an individual’s spatial and temporal gait pattern. Gait characteristics such as step length and step time can be recorded at millimetre and millisecond resolution, respectively [21]. Alternatively, wearable inertial measurement units (IMUs) are finding more routine use due to their greater affordability (compared to instrumented walkways) and ability to be used in a range of environments. IMUs provide equally precise, high-resolution data on a person’s gait with the capacity to provide many more characteristics when fused as part of a sensing array [22]. Accordingly, wearable IMUs are a more contemporary tool for performing accurate gait analysis [23].

Often, IMUs have routine connectivity capabilities through the Internet of Things (IoT), where they can routinely integrate with other systems, offering a streamlined and more rounded experience for clinicians and patients alike [24]. IMUs are also capable of real-time sensing. That is useful by providing users with precise gait data with the immediate delivery of some feedback mechanism(s) during a rehabilitation process. Real-time sensing offers clinicians and patients valuable insights into the efficacy of rehabilitation strategies which could then be immediately adjusted to optimize gait [25]. In short, IMU-based real-time monitoring with immediate feedback describes a process of biofeedback, enriching the therapeutic landscape and enhancing patient outcomes further.

## 3. Personalising Interventions

### 3.1. Biofeedback

Biofeedback has gained prominence as an effective method to enhance biomechanical and physiological aspects of movement and, in particular, the gait of those with mobility issues [23]. Biofeedback offers a real-time response to various physiological signs such as body movement and cardiovascular parameters, allowing patients to gain a better understanding of their physical condition, which is beneficial for those who find it difficult to recognize their physical limitations [26].

Moreover, biofeedback plays a significant role in neural plasticity, activating neural circuits vital for motor control and learning [27]. This can be transformative for individuals with mobility issues, enabling them to achieve meaningful improvements in overall function. Ultimately, biofeedback aids immediate rehabilitation which can contribute to long-term independence and improved quality of life for patients [28].

Real-time biofeedback suggests personalised interventions, i.e., tailored to an individual’s physiological condition. This can enable a more nuanced approach to address the physical and cognitive aspects of specific mobility challenges, rather than adopting a one-size-fits-all approach. In contrast, contemporary rehabilitation lies in the personalised approach to interventions, where a prominent example is in the implementation of biofeedback through personalised cueing [29].

### 3.2. Personalised Cueing

Cueing techniques play a crucial role in the context of reducing falls by assisting individuals to improve their step-by-step gait patterns and therefore their overall walking stability. Generally, cueing involves providing external stimuli or cues to guide and enhance motor control and coordination during gait [30]. In gait rehabilitation, standardised cueing techniques have been used. For auditory stimuli, these include metronome cues or the use of music, where individuals synchronize their steps to a rhythmic beat, or visual cues such as lines or markers on the floor to encourage specific stride lengths or step widths [7]. These standardized cues provide a consistent framework for gait retraining and can be effective in promoting immediate improvements in gait characteristics [8].

To optimise the efficiency of cueing, researchers have examined personalised cueing mechanisms by incorporating biofeedback. That approach includes customizing the cues to harmonise with individuals’ distinct gait and mobility traits, thereby amplifying their efficacy. By adopting a personalised approach, not only can patients’ comfort be enhanced, but also intervention effectiveness. Various studies have underscored the substantial benefits of tailoring the delivery of cueing mechanisms to align with individual gait characteristics, resulting in a marked improvement in the effectiveness of each cueing mechanism [31,32].

To enable personalised cueing, the fundamental requirement is the accurate and near real-time recording of gait, which can be performed with IMUs [33]. By leveraging specific gait characteristics, such as cadence, it becomes possible to modify cueing mechanisms to align with the physiological condition of individuals. For example, gait abnormalities have been reduced by the use of a personalised auditory cue (a 10% increase in the number of beats per minute on an individual’s natural cadence) [34]. To further refine personalised gait rehabilitation, sonification is proposed as a dynamic and versatile methodology.

## 4. Sound and Sonification Theory

Fundamentally, sonification is a method that uses sound, i.e., non-speech audio, to convey information or data. This involves processing non-auditory data and converting it into an auditory format, thereby creating an alternative data perception channel [35]. The technique can be useful when visual channels are overloaded or less efficient, or not accessible [36,37]. Several acoustic variables are typically used to represent qualitative, quantitative, and categorical aspects of information, which include pitch, timbre, amplitude, tempo, duration, and spatialization [11,38].

A wide range of sonification techniques has been explored to represent data, including sonic landscapes, auditory icons, ambient soundscapes, tonal music, ad hoc pitch mappings, simple pitch mapping, speech and non-speech audio, earcons, and musical motifs [39]. Like personalised cueing, applying sonification to gait rehabilitation involves utilising gait monitoring technology such as IMUs to provide real-time auditory biofeedback. For example, gait characteristics can be transformed into discernible sounds [40]. Nown et al. [19] have comprehensively explored the various ways sonification has been mapped to specific aspects of movement, including position, velocity, orientation, and acceleration, with auditory feedback ranging from pitch, amplitude, and timbre to more complex melodies and spatial cues. However, there is a lack of focus on how acoustic variables can influence the overall effectiveness of interventions. Thus, there is also a lack of focus on how such acoustic variables can be manipulated to further enhance treatment; this requires further investigation.

However, before exploring the various applications of sonification and auditory biofeedback, it is crucial to have a solid understanding of sound theory. This ensures optimal approaches for comprehending and implementing sonification and its core concepts into gait rehabilitation.

### 4.1. Acoustic Variables

#### 4.1.1. Frequency

**Figure 1:**
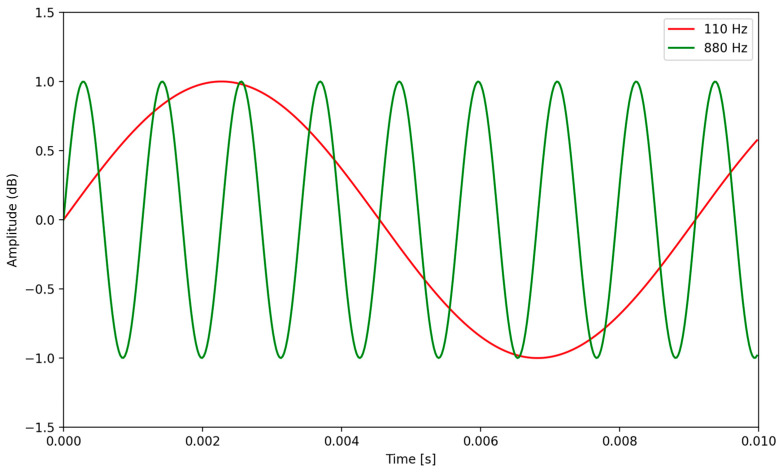
This graph displays the waveforms of musical notes A1 and A5 sampled at a rate of 44.1 kHz. The waveform of the A1 note has a lower frequency of 110 Hz, while the green curve represents the A5 note with a higher frequency of 880 Hz, demonstrating that the ‘closer’ the waves, the higher the pitch.

Frequency is a fundamental property of sound and refers to the number of cycles of a sound wave that occur in one second, measured in hertz (Hz). The frequency of a sound wave ultimately determines its pitch, where shorter frequency wavelengths are associated with higher-pitched sounds and longer frequency wavelengths are associated with lower-pitched sounds. In sonification, the frequency of auditory stimuli can be used to represent various data attributes or dimensions [41]. For example, different data values/variables can be mapped to specific frequency ranges, allowing users to perceive variations in pitch and identify patterns or trends in the data [42]. By assigning frequencies to data parameters, sonification designers can create intuitive auditory representations to enhance the understanding of complex information [43]. Figure 1 illustrates the difference in waveforms with varying frequencies.

#### 4.1.2. Amplitude

**Figure 2:**
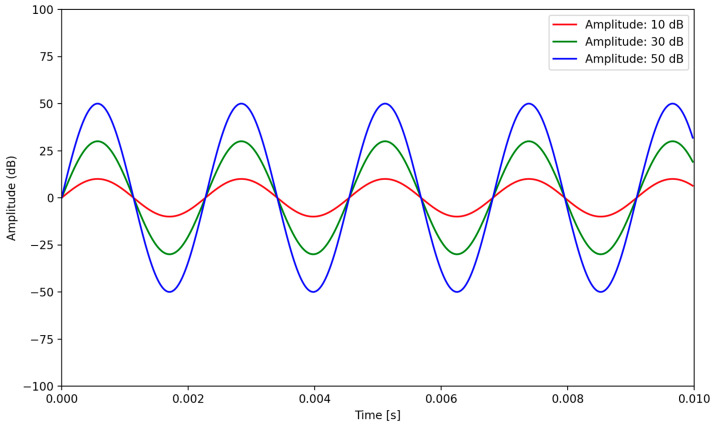
The graph displays three sine waves, each generated at 440 Hz but with different amplitudes of 10 dB, 30 dB, and 50 dB, demonstrating that a higher waveform results in a higher amplitude.

Amplitude represents the strength or intensity of a sound wave and is typically measured in decibels (dB). Amplitude directly affects the loudness or volume, with larger amplitudes producing louder sounds and smaller amplitudes resulting in softer sounds [44]. In sonification, the amplitude can be used to convey the magnitude or intensity of data values, whereby mapping data attributes to specific amplitudes, users can perceive variations in loudness and quickly identify data extremes or significant changes [45]. Figure 2 typifies waveform difference between three amplitudes.

#### 4.1.3. Timbre

**Figure 3:**
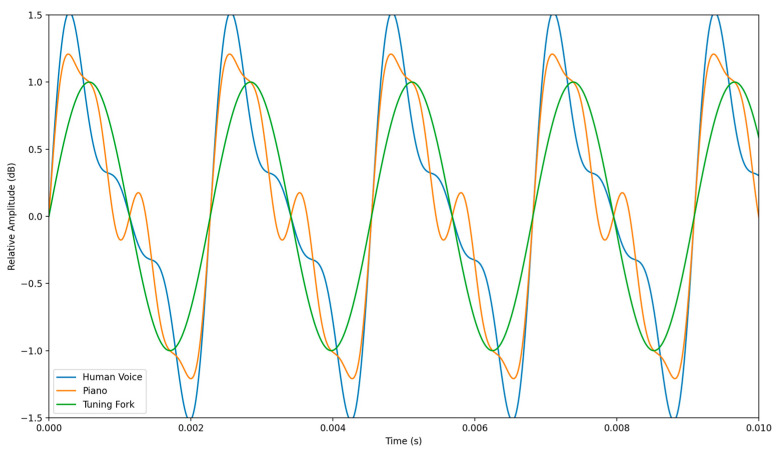
Displays waveforms of 3 audio signals: human voice, piano, and tuning fork, all centred around 440 Hz fundamental frequency. Timbre varies in all due to the difference in unique harmonic structure, seen in each waveform.

Timbre refers to the unique quality or tone of a sound, enabling a distinction between musical instruments or voices producing the same pitch and amplitude [46]. Timbre is influenced by factors such as harmonics, envelope, and spectral content. Harmonics are additional frequencies accompanying the fundamental frequency, giving instruments or voices their characteristic timbre [47]. Envelope represents the dynamic changes in a sound’s amplitude over time, including attack, decay, sustain, and release, and finally, spectral content refers to the distribution of energy across frequencies, contributing to the timbre [48]. For sonification, timbre can represent distinct data categories or attributes, aiding the differentiation of information through auditory characteristics and enhancing the identification of data dimensions [43]. Figure 3 as an example shows the difference in waveforms between a human voice, piano, and tuning fork.

With these acoustic variables and their potential use within sonification being established, it is essential to understand how they can be used to enhance auditory rehabilitation.

### 4.2. Manipulating Acoustic Variables

#### 4.2.1. Time Stretching

Several methods of acoustic manipulation have been explored. One prominent method is the application of a phase vocoder to perform time stretching, which has opened possibilities in gait training [49]. The phase vocoder provides the ability to modify the tempo of music while preserving its original pitch, paving the way for a new level of personalised auditory experiences. Time stretching through the use of the phase vocoder algorithm employs a multi-step process [50] where, first, the original audio signal is divided into short segments using an analysis window, such as a Hanning Windows, and Fourier transforms are subsequently applied to obtain the short-time Fourier transform (STFT) representation. In the modification step, the amplitudes of the sinusoidal components in the STFT are adjusted according to a modification factor, while the phases are calculated using phase unwrapping and a phase-propagation formula to ensure coherence.

**Figure 4:**
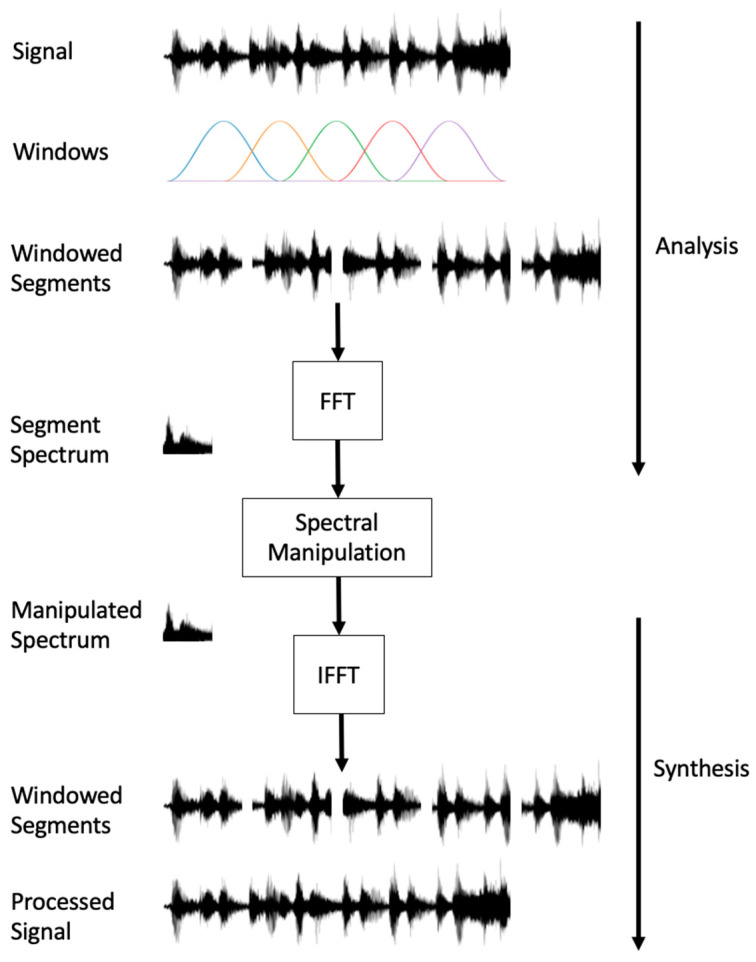
A diagram outlining the steps commonly taken during the phase vocoder algorithm for pitch shifting and time-stretching purposes.

Finally, in the resynthesis stage, the modified STFT is inverse Fourier transformed to obtain short-time signals, which are then combined to create the time-scaled output signal. Although the output signal may not precisely match the original signal’s STFT representation due to the modifications, it retains the local spectral characteristics, which in this case is pitch. Figure 4 below demonstrates the various steps as part of the phase vocoder algorithm for both time stretching and pitch shifting.

One particularly prudent application of a time-stretching algorithm is the application of personalizing auditory stimuli to match an individual’s real-time gait cadence. For example, as an individual walks, the tempo of the music can adjust dynamically, synchronizing with the person’s stride in real time [49]. However, providing the ability to adjust music tempo without changing the pitch of the music could therefore negate any effect changing the tempo of music can have on gait initiation, motivation, and processing of information.

#### 4.2.2. Pitch Shifting

Conversely, as it has been found that a higher pitch can have a positive impact on the effectiveness of auditory stimuli, it may also be beneficial to modify the pitch of music while maintaining the original tempo. The phase vocoder algorithm can also be used for this purpose, where the algorithm applies a similar framework for pitch shifting as with time stretching, but the difference lies in the processing step after the STFT is applied. Rather than altering frame spacing, the algorithm shifts the frequency components of each frame, which is typically achieved by multiplying each frequency bin by a pitch ratio. However, directly shifting frequencies could lead to phase inconsistencies. Therefore, the phase vocoder again must adjust the phase to ensure smooth transitions between frames [51].

Applying pitch shifting to personalised music cues could enable the creation of personalised auditory stimuli tailored to individual needs or preferences. For instance, the pitch of a music piece could be subtly modulated to enhance emotional engagement with the method of rehabilitation, with the goal being to improve its effectiveness.

#### 4.2.3. Dynamic Range Compression (DRC)

DRC is another audio processing technique that modulates the dynamic range, the disparity between the loudest and softest parts, of an audio signal. By subtly diminishing the amplitude contrast, this technique encourages sonic harmony and balance, ensuring that every element of the sound can be heard clearly and resonantly [52]. The DRC algorithm is carried out in several steps, where first it calculates the amplitude of the incoming audio signal. Then, based on a set threshold and ratio, it determines how much the signal’s amplitude exceeds the threshold and applies gain reduction proportional to this excess. The ratio determines the degree of compression, i.e., a higher ratio results in stronger compression. For instance, with a ratio of 4:1, for every 4 dB the input level increases above the threshold, the output level will only increase by 1 dB.

The threshold level, ratio, attack time, which is how quickly the compressor starts to work, and release time, which is how quickly the compressor stops working after the signal drops below the threshold, are adjustable parameters in the DRC algorithm [53]. This technique could be beneficial for use within auditory biofeedback for several key reasons. First, if the rhythmic aspect of the biofeedback is less distinct, with the use of DRC, percussive components like drumbeats become more pronounced and discernible.

**Figure 5:**
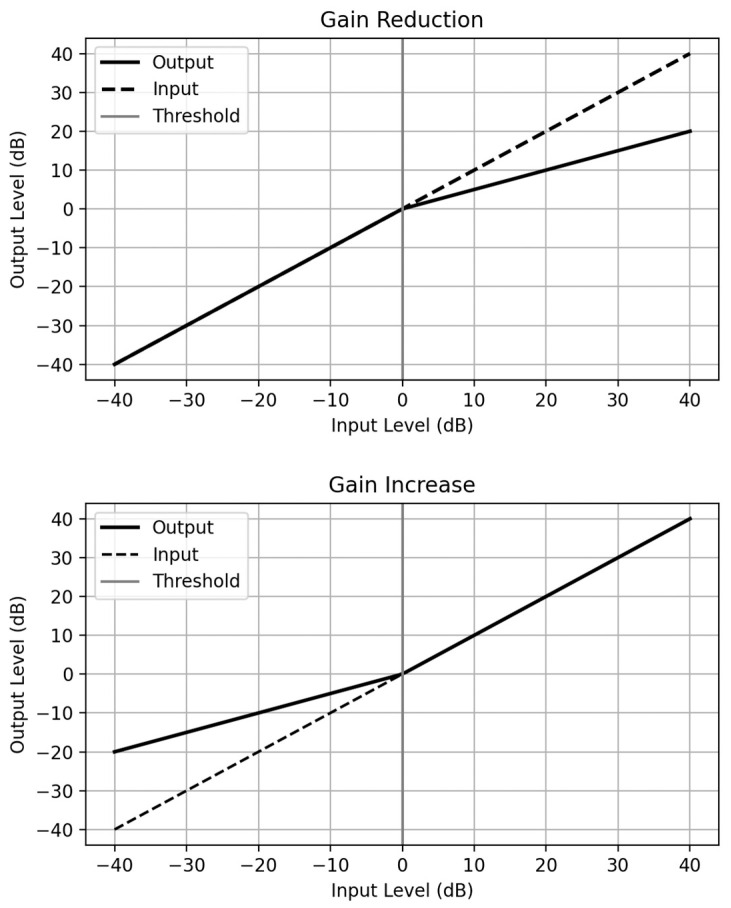
A diagram illustrating the DRC algorithm and how it is used to increase and decrease gain on audio input based on a set threshold.

This improvement could enable patients to synchronize their movements more effectively with the rhythm, facilitating a more immersive and engaging therapeutic experience. Furthermore, its ability to minimize sudden and drastic changes in volume could limit the level of disorientation or distress for individuals undergoing therapy. By applying DRC, such jarring shifts in volume could be reduced, resulting in a more predictable and comfortable listening experience for patients. Figure 5 below illustrates the DRC algorithm being used to increase/decrease the level of gain using a threshold.

### 4.3. Influence of Acoustic Variables on Auditory Rehabilitation

#### 4.3.1. Pitch

To investigate the influence of pitch, a study evaluated the effects of pitch on participants’ emotional response to audio during movement-based tasks [54]. The study uncovered that higher pitch frequencies not only elevated participants’ sense of capability but also boosted their motivation and comfort during physical movement. In contrast, lower pitch frequencies generally produced the opposite effects. The study found also that higher pitches positively influenced valence—the emotional response to pleasantness in external stimuli. This is a significant discovery, given that valence plays a crucial role in initiating gait [55]. Additional research has also discovered that pitch variations also play a role in affecting the perceptual dimension of human cognition, where it critically influences factors such as motivation and the processing of information [51].

#### 4.3.2. Amplitude

To assess the role that levels of amplitude have on auditory gait rehabilitation, a review was conducted on a total of 13 studies that were found through an extensive search [56]. These studies revealed that high-intensity auditory cues significantly enhance gait performance in PD patients, improving metrics like speed, stability, and muscle activation. One study in particular involved 13 PD participants where they were instructed to perform a quick walk initiated with their right leg, taking three consecutive steps in response to three distinct types of stimuli: a visual cue (a white square shown on a black computer screen), a combination of visual and low-intensity auditory cues, and a combination of visual and high-intensity auditory cues [57]. Each auditory cue consisted of 750-Hz tone bursts lasting 30 ms with an amplitude of 80 dB for low-intensity stimuli and 130 dB for high-intensity stimuli. The results of this study revealed that in all PD participants, tibialis anterior and rectus femoris muscle activation was faster with high-intensity auditory stimuli than with low-intensity auditory stimuli. These results suggest that for an auditory stimulus to be effective, a higher level of amplitude may be necessary.

#### 4.3.3. Timbre

Focusing specifically on the effect of timbre, a study involving 13 young adults with diplegic cerebral palsy examined the effect of simple vs. complex chords as auditory cues on gait [58]. Participants were divided into two groups based on the complexity of chords and melodies, where both groups showed significant improvements in walking speed, stride length, and cadence. It was also found that complex chords led to better ankle movement, suggesting that timbral qualities could enhance the effectiveness of auditory cueing in improving mobility for neurological conditions. It is hypothesized in this paper that this is also due to the increased valence in more complex auditory stimuli, demonstrating how timbre can also impact valence and effectiveness.

The evidence indicates that acoustic variables play a crucial role in the success of auditory rehabilitation. By carefully choosing and manipulating auditory cues, it is possible to substantially improve the emotional experience associated with movement. This, in turn, could pave the way for more effective strategies in sonification-based gait rehabilitation, with the sonification of gait characteristics being a key example.

## 5. Sonification as a Gait Rehabilitation Tool

**Figure 6:**
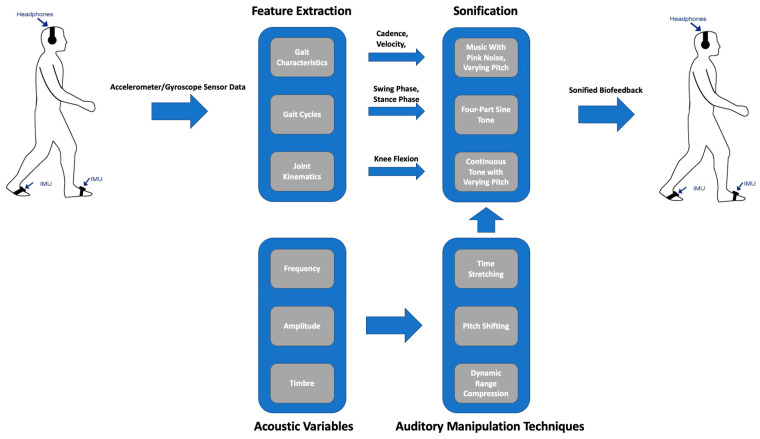
This diagram provides examples, referred to in text, of how sonification and auditory manipulation techniques could be applied to gait rehabilitation, illustrating the extraction of various features from inertial measurement units (IMUs) on the feet. The IMU-based gait characteristics could undergo sonification to generate biofeedback acoustic variables through specialized auditory techniques, thereby changing the person’s gait.

This section explores the various applications of sonification in gait rehabilitation, covering the use of sonified cues generated from various aspects of gait including characteristics, cycles, and joint kinematics. Figure 6 provides a visual summary, covering both the previously discussed topics and the applications of sonification in gait rehabilitation that we will explore in this section. 

### 5.1. Sonification of Gait Characteristics

#### 5.1.1. Cadence Sonification

The application of cadence as a parameter for biofeedback extends beyond personalised cueing, as it has also been explored within the realm of sonification [46]. To explore the potential of utilizing cadence with sonification as a gait retraining approach, a comprehensive study was conducted involving the design and evaluation of a music-sonification system [17]. The first part of the study explores the selection of these feedback signals and the establishment of a perception curve, where the feedback signals tested include white noise, pink noise, amplitude-modulated noise, downsampling, and volume decrease. The tests were conducted on a group of 10 participants who were asked to indicate the perceived intensity level of the feedback signal and asked to fill in a questionnaire to evaluate the clarity, valence, and annoyance of the feedback signals. A statistical evaluation of perception tests involved fitting different curves (linear, second order, exponential) to the data. The accuracy of mapping from objectively measured intensity to subjective perception determined auditory input performance. It was found that pink noise with an exponential fit emerged as the optimal choice, delivering the most perceivable signal and effective mapping, while also having high scores for clarity and pleasantness, solidifying its selection for subsequent experiments.

For the second part of the study, the chosen pink noise was tested under running conditions to determine the minimum perceived sonification level and the just-noticeable difference between different intensity levels. The participants were informed to run while music with superimposed randomized pink noise intensity levels played; the experiment resulted in an interval size of 20% being chosen for further experiments due to the high probability of the detection of a difference in pink noise intensity level.

**Figure 7:**
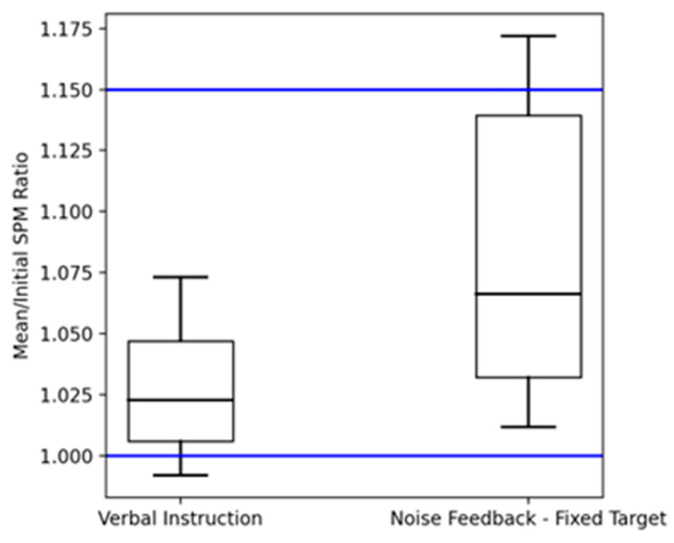
This box plot illustrates the distribution of mean/initial steps per minute (SPM) ratios for two conditions: verbal instruction and noise feedback—fixed target. The blue lines indicate the SPM ratio (1) and the target SPM ratio (1.5).

The study’s final part aimed to confirm the effectiveness of the sonification system in modifying the running cadence of trained athletes. Four conditions were examined: baseline, verbal instruction, fixed target noise feedback, and changing target noise feedback. The baseline measured the average runner cadence post-warm-up, while in the verbal instruction, runners were told to increase their cadence by 15% from the baseline. For the pink noise feedback, runners were asked to adjust their cadence to improve the sound quality of the music they were listening to, which was distorted according to their cadence. Real-time data from two tri-axial accelerometers, one being placed on each leg, were processed to dynamically adjust the music being played to match the runner’s cadence using a phase vocoder time-stretching algorithm. Before the task, runners chose songs from music genres like pop, rock, and dance, with tempos between 140 and 190 BPM, and during the running, if the cadence mismatched the BPM by over 4% for 8 s, a different song played. In the fixed target noise feedback, cadence was tracked and mapped from 95% to 115% of the initial cadence, converted into an exponential curve, and segmented into 20% intervals. The resulting value determined the pink noise level added to the music, with 0% being no noise and 100% being as loud as the music. The changing target phase used similar noise mapping but with fluctuating cadence ranges: 95–115%, 120–85%, and 80–100%. The results, illustrated in Figure 7, found that the pink noise feedback strategy was found to perform significantly better than verbal instructions, suggesting potential for its use in self-training and gait retraining programs, without requiring external assistance. Furthermore, the pink noise feedback was effective for both increasing and decreasing cadence, where feedback from questionnaires also indicated that the combination of minimized noise and synchronized music had a rewarding effect.

#### 5.1.2. Velocity Sonification

Velocity is another gait characteristic that has been explored for gait rehabilitation purposes specifically concerning ameliorating freezing-of-gait (FoG) in PD patients [59]. An experimental protocol, Action Observation plus Sonification (AOS), was devised and applied in a study involving 37 PD patients with bouts of FoG, who were split into two distinct groups: experimental and control. The core experiment involved exposure to eight videos, each demonstrating an actor executing eight unique motor gestures. Participants across both groups were required to mimic these motor gestures synchronously while viewing the videos. However, the marked differentiation between the two groups lay in the sensory aids incorporated into their respective videos. For the experimental cohort, the visual stimuli were supplemented with enhanced auditory signals, which involved the sonification of the walking velocity of the actors. These signals were recorded using a seven-camera motion-capture Qualisys system (120 Hz) and were transformed into audio pitch variations [60]. In contrast, the control group was provided with the same motor gestures, but their videos were complemented by strategic visual (stripes on the floor) and auditory (metronome) cues. For the experimental cohort, a specialized sonification process was employed to generate the enhanced auditory signals from the walking velocity data.

This process first involved the application of a median filter that incorporates a window size of three frames, which suppresses any sensor noise that may potentially originate from the kinematic data. Following this noise suppression, the data stream is adjusted, undergoing a linear scaling process that normalizes it within an interval from 0 to 1. Following this, the generation of the pitch sound itself is executed and subsequently and carefully mapped to the audio channels, distributing it to either the left or the right channel.

**Figure 8:**
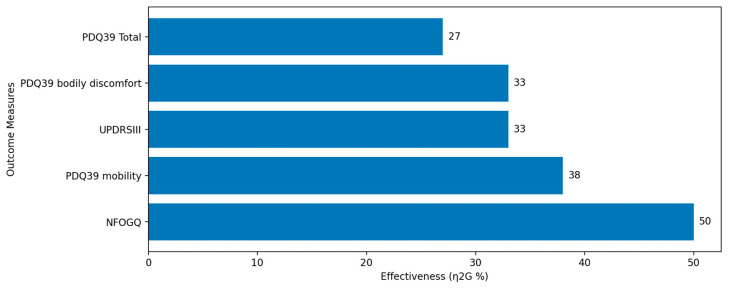
Eta Squared (η2G%) values for the top five outcome measures. The bar chart represents the η2G% for the New Freezing of Gait Questionnaire (NFOGQ), Parkinson’s Disease Questionnaire-39 mobility domain (PDQ39 mobility), Unified Parkinson’s Disease Rating Scale Part III (UPDRSIII), PDQ39 bodily discomfort, and PDQ39 total. Notably, NFOGQ exhibits the highest η2G% value.

The results, illustrated in Figure 8, from the experiment suggest the AOS treatment significantly outperformed the standard cueing protocol, where reduced severity and duration of FoG were observed, not just post-treatment, but also during subsequent follow-ups. AOS also enhanced motor function, reduced daily activity discomfort, and maintained these improvements over time. Additionally, it substantially ameliorated daily living issues and somewhat improved motor balance, with enduring benefits observed three months post-treatment. These results overall showcase the transformative power of the sonification of gait characteristics, underscoring its superiority over traditional metronome cueing in terms of sustained clinical effectiveness. However, while the sonification of gait characteristics has demonstrated effectiveness, the sonification of gait cycles has also been explored as a clinical method of regulating gait.

### 5.2. Sonification of Gait Cycles

A gait cycle, comprising roughly 60% stance phase and 40% swing phase, represents the movements in a single step. The stance phase involves weight-bearing, starting with heel contact (HC) and progressing through loading, midstance, terminal stance, and pre-swing stages. The swing phase, on the other hand, includes the foot’s off-ground movement in stages of the initial, mid-, and terminal swing [61].

The application of sonification for the swing phase of the gait cycle has been investigated to evaluate the efficacy of the approach for delivering effective gait rehabilitation to individuals with PD [18]. Each of the nine PD participants was guided to undertake two full walks, both up and down, along a designated 12-m pathway, where a total of five reflective markers (200 Hz) were attached to the shoes of each participant. Positional (X and Y axes) data were streamed from the markers during each walk, with the sonification of each swing phase being calculated and subsequently played in real time. This sonification process first involves each X and Y displacement alteration consolidated into a singular vector for each heel marker, where the absolute variance in displacement between two distinct samples is calculated to provide an approximate measure of velocity. The commencement of the swing phase of the step is defined when this calculated velocity exceeds a predetermined threshold of 0.5 ms^−1^, which then subsequently triggers the onset of the sonification synthesis process. This process involves a four-part sine tone being played, starting with a fundamental frequency of 261.63 Hz.

**Figure 9:**
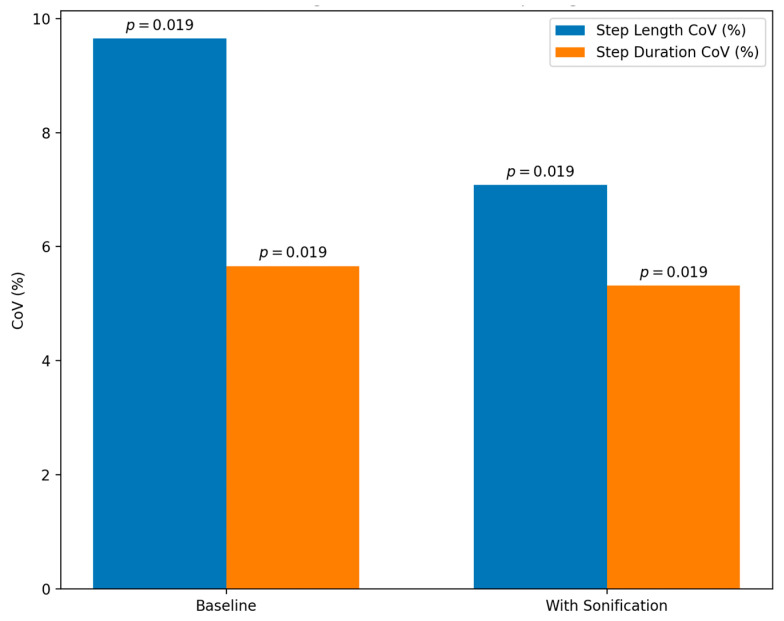
A bar chart illustrating the results of the experiment, demonstrating the significant effectiveness of sonification in increasing step length.

As the distance from the initial stride increased in 0.1 m increments, each subsequent tone was played, with the pitch rising by 1 MIDI number per interval, which creates an auditory sliding effect for each stride. The sonification of each swing phase for each respective foot was subsequently played in the corresponding ear of the individual. The results, illustrated in Figure 9, from the study showcase that when listening to the sonified swing phases while walking, the coefficient of variation (CoV) for step length decreased to 7.08% from 9.65%, while the CoV for step duration decreased slightly to 5.32% from 5.66%. The decrease in CoV for step length was statistically significant (*p* = 0.019), indicating a meaningful improvement compared to baseline measurements. However, the change in CoV for step duration was not significant (*p* = 0.675). These findings suggest that the real-time sonification of gait cycles, i.e., the swing phase, can significantly reduce spatial variability in the gait of PD patients, potentially improving gait consistency.

The promising outcomes of sonifying gait characteristics and cycles highlight the potential of sonification within gait rehabilitation. Building upon these findings, the next section delves into another aspect of sonification in the field of biomechanics, focusing on the sonification of joint kinematics during ambulation.

### 5.3. Sonification of Joint Kinematics

Joint kinematics refers to the study of joint movements and the measurement of angles at specific joints during different activities, such as walking. One specific area where joint kinematics and rehabilitation intersect is the rehabilitation of knee flexion, where the use of sonification has been explored for this purpose [62]. The development of a novel prototype system, namely Sofigait, was conducted to both address the necessity of quantifying knee flexion, as well as to provide sonification to evaluate the perception of sonification feedback in participants with artificially created gait asymmetry. This system, equipped with IMUs and a sonification module, was validated against a Vicon motion capture system in a controlled experiment involving a cohort of 24 healthy individuals.

For the first phase of testing, participants walked on a treadmill at a speed of 4 km/h while their gait was recorded by Sofigait and Vicon. Ten gait cycles captured by both systems were compared for measurement system analysis. For the second phase of testing, participants wore a knee brace on their right leg to create an artificial gait asymmetry and walked five times consecutively on the treadmill at a reduced speed of 3.5 km/h to compensate for the gait restriction caused by the knee brace. They received sonification through wireless headphones from the Sofigait system, which was randomized into four different versions for trials 2–5, where trial 1 served as an acclimatization trial. Throughout each of the trials, the sagittal knee angle is systematically measured and sonified with the position of an extended leg serving as the reference point, denoted as 0°. Subsequently, as flexion increases, the knee angle progressively rises, eventually reaching 180°, which signifies the angle formed between the thigh and lower leg segments.

Four distinct versions of sonification, Soni 1.1, 1.2, 2.1, and 2.2, were implemented as part of the sonification module. Soni 1.1 utilizes a sine continuous tone during the swing phase, ranging from 35°. The knee angle is logarithmically mapped from −45° to +90°, resulting in frequencies spanning from 220 Hz to 1760 Hz. Notably, at 0°, the knee angle corresponds to the concert pitch of 440 Hz, effectively modulating a sine oscillator. Additionally, the knee angle is mapped to the volume, gradually fading out linearly below 35° until it reaches 0°.

Soni 1.2 employs the same principles as Soni 1.1 but with a lower pitch, half an octave below. For Soni 2.1, a continuous sine tone is utilized during the stance phase, ranging up to 35°. Like the previous versions, the knee angle is mapped to the volume, but in this case, the continuous tone gradually decreases linearly from 0° to 35°. As a result, an alternating left/right sound is generated, with emphasis on the smaller angles. Lastly, Soni 2.2 shares the same principles as Soni 2.1 but with a half-octave lower pitch. Table 1 below summarizes the principles of each Soni version.

The results of the study showed that changes in pitch significantly affected the perceived pleasantness of the sonification feedback, with participants indicating that a lower pitch was more pleasant. However, the accentuation of the feedback did not appear to influence participants’ perception of their gait asymmetry. Based on the findings, the swing-accentuated and lower-pitched version of the sonification (Soni 1.2) received the highest ratings on both the asymmetry perception and pleasantness and is, therefore, the recommended approach from the four sonification techniques for patients with asymmetric gait from restricted knee movement.

While these results are limited regarding their effectiveness in reducing asymmetry, they further indicate the potential impact that acoustic variables such as pitch can have on the pleasantness of sonification as a clinical intervention. This finding also emphasizes the requirement for advanced audio processing techniques to effectively address any potential negative impact that acoustic variables, such as pitch, timbre, and amplitude, may have on sonification and personalised cueing in general. Table 2 provides a summary of methods and results regarding gait sonification.

### 5.4. The Unmet Clinical Need

As evidenced in a review by Nown et al. [19] and in this paper, sonification has rehabilitation potential in movement and gait. However, there is a clear lack of focus within the literature on the sonification of clinically relevant gait characteristics that comprise five gait domains: pace, rhythm, variability, asymmetry, and postural control. This is important as these domains are associated with specific cognitive domains: attention, working memory, visual memory, executive function, visuospatial function, and global cognition [63,64]. Those identified gait domains are not uniform but rather involve different neural mechanisms, where, for example, attention has been linked with gait velocity and with grey matter volumes in certain brain regions [65]. Such gait domains become even more distinct in the context of pathological gait as they specifically each have connections to neurodegenerative diseases (such as PD) and general cognitive decline [66,67].

Accordingly, there is an unmet need for harnessing the use of sonification within clinically defined gait. There is a need for targeted and personalised sonification within specific gait domains and associated characteristics. Subsequently, future research could better enable clinicians to focus on specific aspects of gait to target underlying neural mechanisms and this should be the approach taken going forward.

## 6. Conclusions

Personalised cueing with sonification may have added value in gait rehabilitation. Sonification has demonstrated a myriad of applications in gait rehabilitation, to improve spatial and temporal gait characteristics, gait cycles, and joint kinematics. Research has demonstrated extremely promising results, with sonification-based interventions demonstrating significant improvements in reducing freezing-of-gait, spatial variability, and motor balance, and enhancing gait consistency in individuals with mobility disorders such as PD.

Moreover, our comprehensive exploration of sound theory and manipulation of acoustic features, including aspects such as pitch, timbre, and amplitude, has highlighted the significant potential to augment the effectiveness of auditory-based gait interventions. Through detailed analysis, we have discussed and shown how these techniques can profoundly impact factors such as valence, motivation, and information processing. This understanding not only underpins the physical effectiveness of these interventions on improving gait but may also demonstrates their practical application, which is imperative for long-term engagement. The insights gained from this discussion reinforce the importance of these auditory elements, showing how they enhance the overall efficacy of the interventions, thereby offering promising avenues for future research and application in neurological rehabilitation.

Overall, the application of sonification and the manipulation of acoustic biofeedback in gait rehabilitation has the potential to improve personalised interventions and improve the effectiveness of therapy to reduce falls. By providing real-time auditory feedback and tailored auditory experiences mapped to gait domains and clinically relevant characteristics, sonification-based interventions could enhance motor learning and promote neural plasticity. Such an approach could ultimately contribute to improving mobility and reducing falls, restoring independence and improving the quality of life.

## Figures and Tables

**Table 1:** Summary of Soni version principles.

Attribute	Soni 1.1	Soni 1.2	Soni 2.1	Soni 2.2
Phase of Action	Swing Phase	Swing Phase	Stance Phase	Stance Phase
Tone Type	Continuous Sine	Continuous Sine	Continuous Sine	Continuous Sine
Angle Range	35° to −45° to +90°	35° to −45° to +90°	Up to 35°	Up to 35°
Frequency Range	220 Hz to 1760 Hz	Lower by half an octave (165 Hz to 1320 Hz)	220 Hz to 1760 Hz	Lower by half an octave (165 Hz to 1320 Hz)

**Table 2:** Summary of gait sonification methods and outcomes.

Approach	Method	Population	Main Outcomes
Cadence Sonification [17].	A music-sonification system with pink noise, runs under various conditions. Real-time adjustment based on the runner’s cadence.	10 healthy participants	Pink noise feedback significantly better than verbal instruction for altering cadence. Effective for both increasing and decreasing cadence.
Velocity Sonification [59].	Action Observation plus Sonification (AOS) using visual and enhanced auditory cues.	37 PD patients	Reduced severity and duration of FoG. Improved motor function and daily activity comfort. Lasting benefits observed.
Sonification of Swing Phase [18].	Real-time sonification of the swing phase of the gait cycle with specific pitch tones.	9 PD patients	Statistically significant reduction in the CoV for step length. Improved gait consistency.
Sonification of Knee Flexion [62].	Prototype system Sofigait for sonification during walking. Different versions of sonification techniques.	24 healthy individuals	Lower pitch (Soni 1.2) was found to be more pleasant. Limited data on the effectiveness in reducing gait asymmetry.

## Data Availability

No new data were created or analysed in this study. Data sharing is not applicable to this article.
